# Gene changes may minimize masculinizing and defeminizing influences of exposure to male cotwins in female callitrichine primates

**DOI:** 10.1186/s13293-016-0081-y

**Published:** 2016-06-02

**Authors:** Jeffrey A. French, Brett Frye, Jon Cavanaugh, Dongren Ren, Aaryn C. Mustoe, Lisa Rapaport, Jennifer Mickelberg

**Affiliations:** Callitrichid Research Center, Department of Psychology, University of Nebraska at Omaha, Omaha, 68182 NE USA; Department of Biology, Clemson University, Clemson, 29634 SC USA; Zoo Atlanta, Atlanta, GA 30315 USA

**Keywords:** Sexual differentiation, Twinning, In utero, Masculinization, Defeminization, Marmoset, Anti-Müllerian hormone, Sex steroid biosynthesis

## Abstract

**Background:**

Sexual differentiation in female mammals can be altered by the proximity of male littermates in utero, a phenomenon known as the intrauterine position effect (IUP). Among simian primates, callitrichines (marmosets and tamarins) are likely candidates for IUP, since they exhibit obligate dizygotic twinning and fetuses share extensive vascularization in utero. In this paper, we determined whether female reproductive parameters are altered by gestating with a male twin and evaluated changes in genes associated with anti-Müllerian and steroid hormones in twinning callitrichine primates.

**Methods:**

We assessed the impact of gestation with male cotwins on reproductive performance and survivorship in female marmosets (*Callithrix*) and lion tamarins (*Leontopithecus*), contrasting measures for females gestated with one or more littermates (M+) or no male littermates (0M). We compared targeted coding regions for genes involved in steroidal and anti-Müllerian hormone mediation of sexual differentiation for representatives of twinning callitrichines (*Callithrix*, *Saguinus*, and *Leontopithecus*) with closely related New World primates that produce single births (*Saimiri* and *Callimico*).

**Results:**

IUP effects in females were absent in female callitrichine primates: age at first ovulation, average litter size, and the proportion of stillborn infants, and lifetime survivorship did not differ between M+ and 0M females. We documented multiple nonsynonymous substitutions in genes associated with steroid synthesis, transport, and cellular action (*SRD5A2*, *CYP19A1*, *SHBG*, and *AR*) and with anti-Müllerian hormone (*AMH* and *AMHR2*) in callitrichines. In the only callitrichine to produce single infants (*Callimico*), two genes contained nonsynonymous substitutions relative to twinning callitrichines (*CYP19A1* and *AMRHR2*); these substitutions were identical with nontwinning *Saimiri* and humans, suggesting a reversion to an ancestral sequence.

**Conclusions:**

In spite of a shared placental vasculature with opposite-sex twins throughout embryonic and fetal development, female callitrichine primates gestated with a male cotwin exhibit no decrement in reproductive performance relative to females gestated with female cotwins. Hence, IUP effects on female reproduction in callitrichines are modest. We have identified mutations in candidate genes relevant for steroid hormone signaling and metabolism, and especially in AMH-related genes, that are likely to alter protein structure and function in the callitrichines. These mutations may confer protection for females from the masculinizing and defeminizing influences of gestating with a male cotwin.

**Electronic supplementary material:**

The online version of this article (doi:10.1186/s13293-016-0081-y) contains supplementary material, which is available to authorized users.

## Background

Sexual differentiation in mammals represents a complex temporal cascade of genetic, endocrine, and neuroendocrine processes. Sexual differentiation toward male phenotypes is associated with the presence of the *Sry* gene, whose protein products result in the development of testes from undifferentiated gonadal tissue [1]. The testes are endocrinologically active early in fetal life, secreting testosterone and other androgenic metabolites, resulting in the masculinization of secondary sexual characteristics, including the brain. The autosomal gene *Sox9* also plays an important role in sexual differentiation, primarily through the upregulation of anti-Müllerian hormone (AMH) production from testicular Sertoli cells [2, 3]. AMH production results in the regression of the Müllerian ducts and their derivatives, including the fallopian tubes, uterus, and upper vagina, suppressing the development of female-typical accessory reproductive structures [4]. A host of environmental variables work in concert with these genetic and endocrine factors to canalize the process of sexual differentiation, and the sex and location of littermates in utero have been identified as important variables that can modulate the process of differentiation. The intrauterine position effect (IUP) is a well-established phenomenon in litter-bearing species, with the majority of research conducted in rodents. Female fetuses that are gestated in closer proximity to male fetuses become partially masculinized, presumably as a consequence of the vascular diffusion of endocrine secretions of male origin via the fetoplacental circulatory system [5, 6].

Both androgens and AMH are active in mediating IUP effects. Androgen-related masculinization of females via proximity to male littermates is supported by both morphological and behavioral evidence. Female rodents that occupy uterine positions between two male fetuses (2M) tend to have masculinized external genitalia and delayed vaginal opening and also exhibit significant delays in puberty, offspring production, and lifetime reproductive performance, relative to females that were not gestated adjacent to male littermates (0M; [5]), although these effects are not universal across species (e.g., [7–9]). Female rodents that gestate between two males also display higher rates of male-typical behavior than females that gestate between two females, including more territorial behavior, enhanced aggression and male-typical sexual behavior in response to low doses of androgens, enhanced spatial memory, and a greater tendency to disperse from natal groups (see review in [5]). The involvement of AMH in IUP-related defeminization is demonstrated by the freemartin syndrome [10] in cattle and other domestic ungulates. Reproductive sterility in females is often a consequence of gestating with a male cotwin, and interfetal transfer of male-derived AMH results in the regression of female accessory reproductive system [11–13]. Thus, there are at least two independent endocrine mechanisms whereby fetal males can alter the timing and trajectories of sexual differentiation in female littermates.

IUP effects have not been evaluated extensively in the simian primates (monkeys and hominoids), since single births are normative for most primates. However, the callitrichine primates (marmoset and tamarin monkeys, family Cebidae) provide a unique test case for IUP effects in anthropoid primates. First, all callitrichine species except one (*Callimico*) have obligate “litters,” producing modal litter sizes of two offspring [14–16], and candidate genes for twinning have been identified [14]. Since callitrichine twins are dizygotic, roughly 50 % of births involve mixed-sex male and female cotwins. Callitrichines also exhibit a host of embryonic, fetal, and fetoplacental features that facilitate the transfer of signaling molecules between cotwins, including hormones involved in sexual differentiation. Following implantation, embryos have a period of delayed development [17], when embryos develop a fused placental chorion and develop extensive vascular anastomoses by day 29 of embryonic development and persist throughout fetal development [18–20]. The efficacy of intertwin transfer via these shared vascular connections is confirmed by the observation that an estimated 95 % of pregnancies that result in twins are chimeric in hematopoietic tissues [21, 22] and possibly other cell lines [23]. Like freemartin cattle, female callitrichines gestated with male cotwins possess cells with both XX and XY chromosomal elements [24–27] as a consequence of the transfer of embryonic stem cells between twins. However, unlike cattle, chimeric female callitrichine primates are not sterile.

Therefore, given the extensive vascular communication between callitrichine twins, one would expect that masculinizing signals from male fetuses are highly likely to be diffused to female cotwins, potentially leading to masculinization and hence reduced reproductive capacity in females. However, only two studies have explicitly addressed this issue in callitrichines, with differing outcomes. Ardito et al. [28] contrasted female reproductive performance in marmosets that possessed both XX and XY lymphocytes (and hence gestated with a male cotwin) relative to females with only XX lymphocytes. A higher proportion of females with XX/XY karyotypes were among the colony’s best reproductive females (measured by number and survivorship of offspring) than XX-only females. In contrast, Rutherford et al. [29] demonstrated that female marmosets that gestated with a male cotwin had a greater lifetime pregnancy loss than females that gestated with a female cotwin, but rates of pregnancy loss were not adjusted for lifetime reproductive tenure.

The present study had two aims. First, we quantitatively assessed the potential reproductive and survivorship costs to females that shared a uterine environment with at least one male in three species of callitrichines, using multiple measures of reproductive performance. We addressed this question by analyzing long-term captive breeding records in female marmosets (*Callithrix* spp.) and golden lion tamarins (*Leontopithecus rosalia*) born in litters with no known male cotwins (0M) and those born in litters with at least one male cotwin (M+). Second, we conducted a comparative analysis of coding regions for candidate genes implicated in sexual differentiation [30, 31] in several genera of twinning callitrichine primates. These included genes that code for aromatase (*CYP19A1*), 5α-reductase (*SRD5A2*), the nuclear androgen receptor (*AR*), the glycoprotein that serves as a carrier molecule for sex steroids (*SHBG*), anti-Müllerian hormone (*AMH*), and the type II anti-Müllerian hormone receptor (*AMRHR2*). We contrasted these sequences with those from the closely related squirrel monkey (*Saimiri*, family Cebidae) and the callitrichine *Callimico*, both of which produce single offspring. If reproduction in female callitrichines is negatively impacted by gestation with a male cotwin, we predicted few large impact nonsynonymous substitutions (NSs) in gene sequences in the twinning callitrichines relative to related species that produce single offspring. However, if female reproductive development and function in twinning callitrichines are not impacted by gestation with a male cotwin, we would expect significant differences in nucleotide and predicted amino acid sequences for these genes in twinning callitrichines in ways that suggest protective mechanisms against masculinization of females that gestate with male cotwins.

## Methods

### Reproductive records

Reproductive performance and offspring survivorship in females born to mixed-sex (M+) vs. same-sex (0M) litters were evaluated for two genera of callitrichine primates: marmosets (*Callithrix* spp*.*) and golden lion tamarins (*Leontopithecus rosalia*). Records for marmosets were derived from the long-term breeding program at the University of Nebraska at Omaha (UNO) and included data on two closely related species: *C. kuhlii* and *C. geoffroyi*. We derived our data for lion tamarins from the international studbook, a digital database on captive breeding in this species. For both marmosets and lion tamarins, females were included in our analyses if breeding records contained information regarding sex composition of litters from which they derived, dates of pairing in a reproductive context, and dates of birth for offspring produced by these females. For each species, the number of females used in the analyses, their distribution as a function of sex composition of the litter into which they were born, and the average number of litters produced throughout their lifetime is indicated in Table 1. We did not genotype 0M females for XX/XY chimerism, and since fetal loss can occur in callitrichines [32], some 0M females may have had exposure to male cotwins at some point in embryonic and fetal development. However, litter-size reduction verified by ultrasound in marmosets has a low incidence (7.2 % of pregnancies; [33]), thus *potentially* misclassified 0M females represent a very small proportion of the overall sample.

**Table 1:** Animal information for reproductive and survivorship analyses

Scientific name	Common name	# Breeding females		Total litters produced		Mean (SEM) litters per female		Institution
		0M	M+	0M	M+	0M	M+	
*Callithrix*	Marmoset	11	12	51	49	4.6(1.0)	4.1(0.6)	Univ. Nebr. Omaha
*Leontopithecus*	Lion tamarin	61	90	282	339	4.6(0.5)	3.8(0.3)	Zoos worldwide

### Reproductive and life-history measures

Our predictor variable for female reproduction was derived from the sex composition of the litter into which breeding females were born. We classified breeding females into all-female litters (0M) or females that had undergone gestation with at least one male fetus (M+). We tested four reproductive parameters to differentiate the effects of litter sex ratios on prenatal and postnatal female reproductive performance. The first three measures evaluate the impact of exposure to male siblings in utero on the development of ovarian function, fetoplacental factors affecting litter size, and the adequacy of gestational ability. *Latency to first parturition*: length of time (in days) between pairing of the female with an unrelated, reproductively intact adult male and first parturition. We excluded pregnancies in females that resulted in preterm birth of offspring, and age at pairing with a breeding male was not available for 13 female *Leontopithecus* (7 0M and 6 M+). *Age at first ovulation*: for a small number of female *Callithrix* (0M: *n* = 6; M+: *n* = 10), we had archived urine samples that allowed us to definitively determine the age at first ovulation based on urinary pregnanediol concentrations. Details on endocrine methods and criteria for first ovulation can be found in [34]. *Litter size*: we calculated the average number of full-term fetuses (both stillborn and alive) produced per parturition event. Females that had given birth to at least one litter were included in this analysis, and litters of all sizes (range = one to five offspring) were included in these calculations. *Proportion of stillborn infants*: we calculated the proportion of the stillborn infants (i.e., offspring that were physically full-term but who were dead upon discovery and showed no signs of maternal abuse or physical damage) from the total number of full-term fetuses produced per parturition event. *Female longevity*: we collected data on age at death for females from both genera and the age of currently living females for the use in a censored survivorship analysis (see below).

### Statistical analyses

For latency to first parturition, age at first ovulation, litter size, and proportion stillborn infants, we contrasted means for 0M breeding females with M+ females using Welch’s *t* test. We used the Cox proportional hazards model to test for differences in lifetime survivorship of 0M and M+ females.

### Genetic analyses

#### Candidate genes

We identified a cluster of genes that have been demonstrated to be critical in the process of normative sexual differentiation in mammals. Four genes are involved in steroid metabolism, receptor function, and transport [31, 35]: *CYP19A1*, a locus coding for the aromatase enzyme that converts C19 androgens into C18 estrogens; *SRD5A2*, which codes for the enzyme 5α-reductase that serves to convert testosterone into dihydrotestosterone; *AR*, which codes for nuclear androgen receptors; and *SHBG*, which codes for sex hormone-binding globulin, a transport glycoprotein that binds to sex steroids and renders them biologically inert. Two genes associated with the early embryonic differentiation of internal genitalia and associated structures were also analyzed: *AMH*, which codes for anti-Müllerian hormone (AMH), a protein that inhibits the embryonic development of female-typical Müllerian ducts that are destined to become uterine structures, and *AMRHR2*, which codes for the type II AMH-receptor that mediates Müllerian duct regression in the presence of AMH.

##### Species

Publically available exomic coding sequences for each gene were aligned firstly across a wide variety of primate species, including human, gibbon, rhesus monkey, common marmoset (*C. jacchus*), squirrel monkey (*Saimiri boliviensis*), tarsier, bushbaby, and mouse lemur; with outgroup species constituting cattle and sheep. All published sequence data were accessed from UCSC Genome Browser, NCBI, or Ensembl. Sequences were aligned in MEGA 6.0 using ClustalW. Regions of the candidate genes that contained *Callithrix*-specific NSs relative to comparison species were selected for further sequence analysis. Specific regions identified by these analyses for each candidate gene and details of PCR amplification are noted in Additional file 1.

We generated de novo nucleotide sequences via traditional Sanger sequencing for the candidate genes in five additional callitrichine species, including *C. kuhlii*, *C. penicillata*, *L. rosalia*, *Saguinus midas*, and *C. goeldii*. The institutional source of tissue or extracted DNA for each species is listed in Additional file 2. We sequenced selected coding regions for the candidate genes from at least two unrelated individuals for each species, one male and one female. Our primary comparisons of interest were (1) the contrast between predicted amino acid sequences for the candidate genes between the twinning callitrichines and the closely related singleton-producing squirrel monkey (*Saimiri*; both members of the Family Cebidae) and (2) between the twinning callitrichine species and *Callimico*, the only callitrichine primate that produces single offspring. Genomic DNA was extracted using the DNeasy Blood and Tissue Kit (Qiagen) following the manufacturer’s protocol. DNA yield from the extractions was quantified using Nanodrop® (Thermo Scientific), and DNA quality was measured using 0.8 % agarose gel electrophoresis. The same sets of PCR primers were used to amplify coding regions in our candidate genes for all species, and the details of PCR protocols are found in Additional file 2. PCR products were purified using QIAquick PCR Purification Kit (Qiagen) and sequenced directly using an Applied Biosystems (ABI) 3730 48-capillary electrophoresis DNA analyzer, in the High-Throughput DNA Sequencing and Genotyping Core Facility at the University of Nebraska Medical Center. Sequences generated de novo for this project are available at NCBI [GenBank: 1882226].

For all New World primates, amino acid substitutions were classified as NSs (and hence potentially relevant for altered protein function) if the nucleotide sequence predicted variable amino acids at each position among species. We further classified NSs as conservative or radical according changes in polarity, charge, and volume: NSs with a change in one or more categories were classified as radical, while NSs with no changes in the three categories were classified as conservative [36]. To estimate the impact of the identified NSs in each gene, we submitted comparative sequences of predicted amino acids in *Saimiri* and the callitrichine primates to an analytical algorithm (PROVEAN; [37]) designed to assess the potential significance of the callitrichine NSs for protein structure, function, and putative phenotypic changes. PROVEAN employs an alignment algorithm to predict the functional impact of protein sequence variations (e.g., single and multiple amino acid substitutions, deletions, and insertions) and can contrast variation between any organisms (in our case, variation between *Saimiri* and *Callithrix*) and is not limited to contrasts with human sequences (e.g., SIFT, Polyphen: [38, 39]).

## Results

### Reproductive performance

#### Gestational/parturient measures

Exposure to a male fetus during embryonic and fetal development had only modest effects on subsequent reproductive performance in female marmosets and lion tamarins (Table 2). Age at first ovulation determined from urinary pregnanediol profiles did not differ between 0M and M+ *Callithrix* females. Overall, female marmosets gave birth to larger litters (2.52 ± 0.15 offspring, mean ± SEM) than female lion tamarins (1.84 ± 0.003 offspring), but average litter sizes did not differ between 0M and M+ females in either species. Pregnancy outcomes were also unaffected by litter sex ratios, as the proportion of stillborn infants across the females’ reproductive lifetime did not differ between 0M and M+ females. For both genera, the latency to first parturition after pairing with an appropriate male breeding partner was affected by litter sex ratio. In Leontopithecus, M+ females took longer to produce their first litter after pairing than did 0M females (*d* = 0.37).

**Table 2:** Reproductive performance in females gestated with (M+) or without (0M) male cotwins. Means (SEM) are provided

Reproductive parameter	0M (SEM)	M+ (SEM)	t
*Leontopithecus rosalia*	*N* = 61	*N* = 90	
Latency to first parturition (days)*^a^	318 (32.9)	421 (34.6)	−2.14*
Litter size	1.84 (0.06)	1.84 (0.05)	0.06
Proportion stillborn infants	0.36 (0.05)	0.37 (0.04)	0.87
*Callithrix* spp.	*N* = 11	*N* = 12	
Age at first ovulation (days)^b^	533 (51.4)	542 (21.7)	0.17
Latency to first parturition (days)	266 (38.0)	381 (54.3)	2.99
Litter size	2.41 (0.16)	2.62 (0.25)	0.49
Proportion stillborn infants	0.24 (0.06)	0.22 (0.09)	0.21

*P < 0.05

^a^ *N*_0M_ = 54; *N*_M+_ = 84

^b^ *N*_0M_ = 6; *N*_M+_ = 10

#### Female lifetime survivorship

**Fig. 1:**
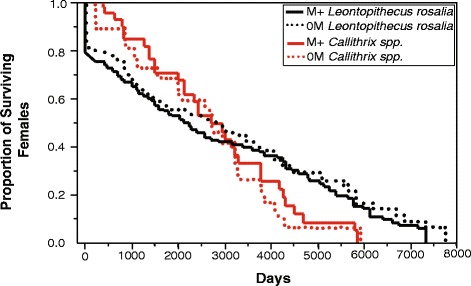
Impact of gestation with male cotwins on female lifetime survivorship and reproductive performance. Lifetime survivorship plots for female lion tamarins and marmosets born with no male littermates (0M) or one male littermate (M+)

Overall lifetime survivorship of females in both taxa was unaffected by the presence or absence of a male cotwin. Figure 1 portrays survivorship curves for females, and the analyses (both censored and uncensored lifetime measures) revealed no significant differences in the pattern of survivorship between 0M and M+ females for either species (*Leontopithecus*: *χ*^2^(1) = 2.38, *p* = 0.12; *Callithrix*: *χ*^2^(1) = 0.11, *p* = 0.74).

### Genetic analyses

**Fig. 2:**
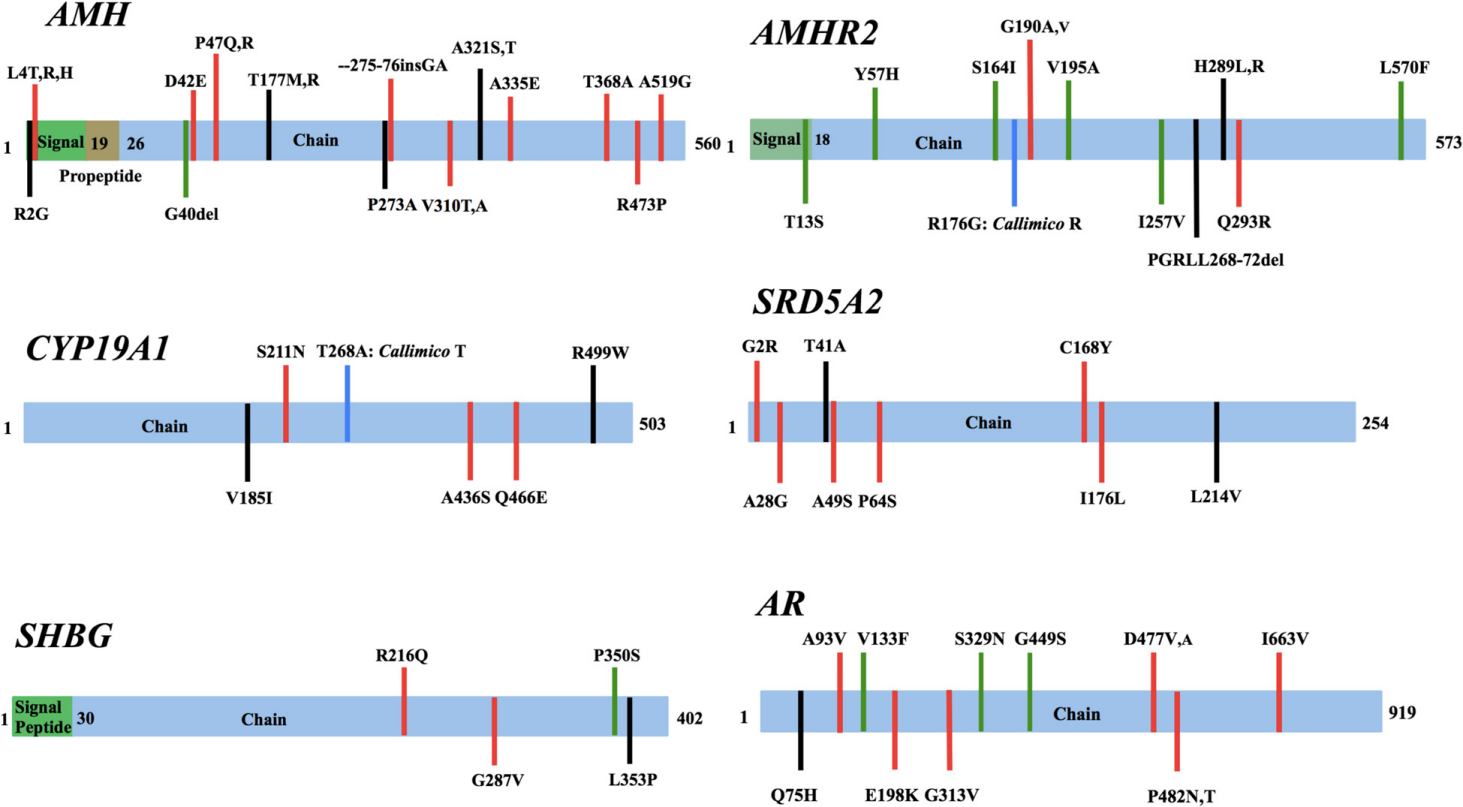
Amino acid changes resulting from nonsynonymous nucleotide substitutions in six genes with prominent roles in sexual differentiation. All substitutions are contrasted with predicted amino acids in the relevant genes from the squirrel monkey, *Saimiri. Red* = amino acid positions in which NSs were present in all callitrichine genera sampled. A single amino acid after the position number indicates the same substitution among all calltrichines; two or more amino acids after the position number indicates multiple residue substitutions in different callitrichine genera; *black* = substitutions in some, but not all, callitrichines; *blue* substitutions in all callitrichines except *Callimico*; *green* = amino acid residues specific to *Callithrix*. Protein regions are based on UniProt annotations. Further details on substitutions can be found in Additional file 3

In the targeted exonic regions of the six candidate genes, we documented 53 NSs in one or more genera of callitrichine primates, relative to *Saimiri*. Substitutions in each gene are presented in Fig. 2 and a summary of these NSs in Table 3. Further details on specific protein positions, amino acid substitutions, and predicted functional consequences across all species sampled can be found in Additional file 3. Of the 53 NSs, amino acid substitutions at 26 positions were documented across all callitrichine genera. Each of the target genes contained at least two callitrichine-wide substitutions (range 2–8). We utilized PROVEAN [37] to identify NSs in callitrichines, relative to *Saimiri*, that were predicted to lead to significant alteration in protein structure/function. We also used SIFT [39] and PolyPhen [38] to identify likely deleterious NSs in callitrichines, relative to protein sequences in humans.

**Table 3:** Summary of nonsynonymous substitutions (NSs) across callitrichine primates in genes relevant for sexual differentiation

Gene	# NSs^a^	All callitrichines	*Callithrix* specific	Variable callitrichine	*Callimico* reversion	% Altered protein^b^	% Radical AA^c^
*AMH*	14	8	1	5	0	21.4	64.3
*AMHR2*	11	1	7	3	1	18.2	80.0
*AR*	10	6	3	1	0	0	80.0
*CYP19A1*	6	3	0	2	1	0	83.3
*SHBG*	4	2	1	0	0	0	100
*SRD5A2*	8	6	0	2	0	12.5	75.0

*All callitrichines* NSs common to all callitrichines sampled, *Callithrix specific* NSs documented only in *Callithrix*, *Variable callitrichine* NSs identified in some, but not all callitrichines, *Callimico reversion* NSs in single-offspring *Callimico* that represent a reversion to *Saimiri* residue

^a^Total #NSs identified in callitrichines

^b^NSs that represent significantly altered protein structure/function by PROVEAN

^c^NSs that lead to at least one physicochemical change in amino acid residue

#### Steroid-related genes

Each of the four genes associated with steroid metabolism, transport, and nuclear activation expressed at least three callitrichine-specific substitutions, and many amino acid positions varied across the callitrichines, relative to *Saimiri*. All amino acid substitutions in *CYP19A1*, *SHBG*, and *AR* were identified by PROVEAN as neutral substitutions with regard to predicted protein structure and function. However, P64S in *SRD5A2* was identified as a substitution that would lead to altered protein structure/function (and was also targeted by SIFT as a deleterious change). We noted one substitution in *CYP19A1* (T268A) that was present in all callitrichines except *Callimico*, and the predicted residue at this location for *Callimico* was T, the predicted residue for *Saimiri* and humans, but this NS was predicted to lead to a neutral change in protein structure/function.

#### AMH-related genes

Amino acid substitutions were more numerous in the AMH-related target genes than in the steroid-related candidate genes. For *AMH*, eight of 14 amino acid positions contained substitutions across all callitrichines. PROVEAN identified three substitutions predicted to significantly alter protein structure/function. These included a deletion (G40del) in *Callithrix* and two substitutions (T177M,R in *Callithrix* and *Callimico*, respectively; R473P in all callitrichines). Although there were only two callitrichine-wide substitutions in *AMHR2* (positions 190 and 293), a deletion at positions 268–272 in *Callithrix* and *Callimico* and H289L in *Callithrix* and *Leontopithecus* was identified as producing significant alterations in protein structure/function. As with *CYP19A1*, a substitution (R176G) in *AMHR2* was present in all callitrichines except *Callimico*, which expressed the same residue as *Saimiri* and human. Relative to steroid-relevant genes, then, NSs were more numerous for AMH-related genes, and these changes were more likely to lead to altered protein structure and/or function.

## Discussion

Given the features of dizygotic twinning and early shared intrauterine vascularization in most callitrichine primates, they would appear to be obvious candidates for the masculinization of females gestated with male cotwins, and consequently, there should be evidence of protection of female fetuses from masculinizing and defeminizing signals from male cotwins. With the exception of latency to produce the first litter, our data reveal that females incur little or no cost to reproduction or survivorship as a result of a shared uterine environment with a male cotwin. These results hint strongly at one or more protective mechanisms in callitrichines that might minimize masculinization and defeminisation in females from mixed-sex litters. In a suite of candidate genes that regulate two important endocrine cascades that mediate sexual differentiation (AMH and androgens), we documented a number of nonsynonymous substitutions in the coding regions for callitrichines that were different from human and macaque monkeys. Importantly, these NSs also differed from the squirrel monkey (*Saimiri*), which provides an important phylogenetically relevant contrast, since *Saimiri* belongs to the same Family (Cebidae) as callitrichines but produces only single offspring.

Latency to produce the first litter after pairing with a male was longer in M+ than 0M females in *Leontopithecus* but not in *Callithrix*, although the absence of a significant effect in *Callithrix* may be a consequence of a relatively small sample size and hence low statistical power. There appear to be species differences in the effects of gestating close to males on the onset and success of female reproduction. In mice, while many features of female morphology and behavior are masculinized among 2M females [5], 2M females do not appear to have delayed conception or reduced offspring survivorship, relative to 0M females [40, 41]. In pigs, however, females born into male-biased litters have higher reproductive failure during their first pregnancy and have reduced reproductive success over their first four litters, relative to females born in female-biased litters [42]. The extent to which delayed conception in M+ callitrichine females represents a common trait among species in this taxon, or is unique to the reproductive biology and life history of *Leontopithecus*, will require analyses characterized by both larger sample sizes and additional species.

Our candidate genes were selected on two premises: (1) both AMH and androgens are critical in the process of sexual differentiation in mammals [1] and (2) mutations in these genes are associated with abnormalities in sexual differentiation, particularly undermasculinization and defeminization, in human populations [30]. In our six candidate genes, we identified a total of 53 NSs leading to a change in predicted amino acids that differentiated one or more species of callitrichines from *Saimiri*, with 21 NSs that were common to all callitrichine genera sampled. *AMH* and *SRD5A2* each had six callitrichine-specific substitutions. *SHBG* had the fewest amino acid substitution of the candidate genes, and only two of the four substitutions were present among all callitrichines. Across genes, the majority of substitutions represented a radical change in the physicochemical properties of the residue [36].

Multiple single nucleotide polymorphisms (SNPs) in androgen-relevant genes have been documented that alter the process of masculinization [43, 44]. Analyses of *SHBG* in female-masculinized spotted hyenas (*Crocuta crocuta*), relative to nonmasculinized hyenid species, reveal multiple substitutions that reduce production of SHBG, thereby increasing bioactive sex steroids in this species [45]. To the extent that protection against masculinization for female callitrichines is mediated partly by modified SHBG, we would expect the NSs to increase either sex steroid affinity or production of the binding protein. SNPs that lead to reductions in efficacy or loss-of-function for enzymes involved in steroid biosynthesis have been documented for both *CYP19A1* [44–46] and *SRD5A2* [47–49], and these changes produce an undermasculinized phenotype in males. Our data revealed a callitrichine-specific NSs in *SRD5A2* at protein position 49 (A49S), and polymorphisms at this position in human alter aromatase activity in bioassay and produce hypospadic phenotypes in males [50–53]. Further, PROVEAN identified a callitrichine-specific amino acid substitution (P64S) in *SRD5A2* that was predicted to significantly alter protein function in 5α-reductase, relative to *Saimiri*. Finally, polymorphisms in the coding region for AR are also known to produce undermasculinization in human [54, 55]. We identified nine NSs in AR across callitrichines, with six common among all genera sampled. However, none of these substitutions were predicted to significantly alter AR structure or function by PROVEAN.

Dysfunction in the AMH signaling system can derive from nucleotide substitutions in both the ligand and the type II receptor genes [4, 56–58]. Our comparative analyses of callitrichines vs. *Saimiri* revealed multiple NSs in these genes, with significantly altered protein function as a consequence of three substitutions in *AMH* and two in *AMHR2*. The clinical condition of persistent Müllerian duct syndrome, in which males exhibit normal external genitalia but possess fallopian tubes and uteri, is associated with mutations in the coding regions for both AMH ligand and receptor genes [59, 60] in regions of exons in close proximity to NSs we identified in callitrichine primates. These clinical findings suggest that AMH system polymorphisms reduce the canalizing of early reproductive development toward a male phenotype, and the NSs we identified represent important candidates for female protection from AMH-mediated masculinization in callitrichines.

There are a growing number of reports of female callitrichines that present with masculinized features, but these reports suggest that the masculinizing effects are limited to external genitalia and are not associated with atypical development of primary gonads or accessory reproductive structures. Multiple callitrichine breeding colonies have reported the occurrence of females born with ambiguous masculinized genitalia, including hypertrophied clitori and congenitally fused labia with a single urethral opening, in contrast to normative urethra and vaginal structures. These atypical females have been reported in both *Callithrix* and *Leontopithecus*. In cases where the sex of the female’s cotwin was reported, it was universally a male, and karyotyping or genotyping of females presenting with atypical genitalia revealed the presence of Y chromosomes in lymphocytes or Y chromosome-associated zinc-finger protein (Y-ZFP) [25, 26]. Endocrine analyses of females indicated normative ovarian function including progesterone profiles consistent with ovulatory cycles [25, 61, 62] and low levels of testosterone [25] and its metabolic precursor androstenedione [62]. Ultrasound or post-mortem analyses revealed female-typical structures in the fallopian tubes, uterine horns and uterus, cervix, and vagina, ovaries that displayed primary and secondary follicles and corpora lutea, and no signs of testicular tissue [24, 25, 61, 63]. In one case in which the fused labia were surgically separated, the female became pregnant and delivered a normal twin litter after pairing with a male [62]. Collectively, these data suggest that while there may be masculinizing effects of a male cotwin on the structure of external genitalia in females, the development of the accessory reproductive system in females is relatively insensitive to the presence of males in utero. Given that the differentiation of accessory reproductive structures is strongly regulated by AMH, this pathway is likely to be more important in protecting female offspring from the defeminizing consequences of exposure to a male cotwin. Our data show that more NSs, and a greater proportion NSs with significant predicted changes in protein structure and function are found in AMH-related genes in callitrichine species, suggesting that this system is has undergone selection in twinning species and hence may serve an important role in female protection against defeminnization in utero.

A proposal has been forwarded regarding a shift from prenatal to postnatal staging of development in callitrichines, including the realm of sexual differentiation [64]. Abbott and Hearn [65, 66] suggested that the majority of differentiation of behavioral sex differences, and some aspects of genital morphology, may occur postnatally as an evolutionary adaptation to minimize the impact of male-derived endocrine signals for masculinization and defeminization. This notion is supported by normative endocrine data, since testosterone levels are elevated in males but not females from 15 to 100 days postpartum [67–69]. Immediate postnatal treatment of females with exogenous testosterone partially masculinizes female external genitalia [66, 70]. Neonatal castration produces adult males with slightly demasculinized genitalia, and testosterone treatment of these castrates as adults results in increased penile and scrotal size [71]. Behavioral sexual differentiation in callitrichines appears to be organized in part by postnatal endocrine factors, since neonatal castration in males is associated with lower rates of sexual and aggressive behavior as adults [72, 73] and neonatal androgen treatment in females enhances male-typical sexual behavior and reduces female-typical behavior [65]. The observation that untreated females gestated with male cotwins showed no male-typical behavior and perfectly competent female sexual behavior is evidence for both the postnatal staging hypothesis and for the presence of mechanisms to minimize masculinization of females in utero. However, marmoset genitalia are typically well-differentiated at birth, and immediate postnatal androgen manipulations only slightly modify genital morphology [70]. Thus, delayed timing in behavioral sexual differentiation, along with NSs-associated altered function in hormones, enzymes, and receptors involved in sexual differentiation of accessory reproductive structures, may work together to minimize masculinization and defeminization of females in twinning callitrichine primates.

*Callimico*, the only callitrichine that produces single-offspring litters, represents an interesting “control” species for our analyses. Recent molecular phylogenies have placed this genus as a sister taxa of *Callithrix* [74, 75], suggesting that *Callimico* does not represent an ancestral callitrichine from which twinning species subsequently evolved but rather a derived species that lost the ability to produce twin litters. *Callimico* shares a number of NSs with the other callitrichines, relative to *Saimiri*. However, we noted two NSs in *Callimico* that led to a reversion to the *Saimiri* and human amino acid residue in AMHR2 and CYP19A1. To the extent that NSs associated with female protection are neutral for single-litter offspring, there is no reason to expect reversion. However, in the event that NSs associated with sexual differentiation in twinning species are deleterious for single-litter species (e.g., reduction in masculinization) then reversions are to be expected. The two NSs we identified thus represent good candidates for further evaluation for critical roles in sexual differentiation and female protection from masculinization/defeminzation.

Our sequence results, while suggesting important differences in the genes involved in the steroid- and AMH-mediated components of sexual differentiation, do not definitively demonstrate that the NSs identified in callitrichines represent the key “protective” factors that minimize female masculinization and defeminization in utero, nor do they imply that they are the only components of the complex signaling cascade the yields male-female differences. Future studies that highlight the full coding and promoter regions of the genes we selected for analysis and additional candidate genes involved in sexual differentiation will provide a more complete list of candidate alleles and NSs that may protect female fetuses in twinning vs. nontwinning simian primates. Further, knowledge of the pattern of expression of our candidate genes and their protein products, both in a developmental context and with regard to tissue specificity of expression between males and females, will assist in understanding the full story of prenatal and postnatal sexual differentiation.

## Conclusions

In sum, our data show that the reproductive and longevity consequences of sharing a common uterine environment and extensive vascular connections with a male cotwin for female callitrichines are minimal, with no effects on female longevity, litter size, number of stillborn infants, or age at the onset of first ovulation. These findings are in marked contrast to the extreme masculinization and sterility in the freemartin condition in domestic cattle and other species [4], with which callitrichines share the common traits of XY chimerism in females and extensive shared vascularization among opposite-sex cotwins. The notion that selection may have favored mechanisms that protect female callitrichines against masculinization and defeminization in utero is supported by the large number of NSs in a suite of genes that are critical for morphological, neuroendocrine, and behavioral sexual differentiation. Our finding that more amino acid substitutions were documented in AMH-related genes than in genes associated with steroid synthesis, transport, and cellular action is consistent with clinical cases and colony-wide surveys of masculinized external genitalia in females that gestate with male cotwins but normative accessory reproductive structures and sex-typical behavior in these females.

### Abbreviations

AMH, anti-Müllerian hormone; AMHR2, anti-Müllerian hormone receptor type 2; AR, androgen receptor; CYP19A1, aromatase; IUP, intrauterine position effect; NS, nonsynonymous nucleotide substitutions; SHBG, sex hormone-binding globulin; SRD5A2, steroid-5-alpha reductase

## Additional files

Additional file 1: Regions of candidate genes sequenced de novo for this study and PCR primers used to amplify selected regions. (DOCX 19 kb) Additional file 2: Tissue or DNA source for gene sequencing. (DOCX 67 kb) Additional file 3: Summary of amino acid residue substitutions and predicted change in structure/function (PROVEAN, SIFT, and PolyPhen) for the six candidate genes for human, *Saimiri*, and four callitrichine primates. (XLSX 19 kb)

## References

[1] Quinn A, Koopman P (2012). The molecular genetics of sex determination and sex reversal in mammals. Semin Reprod Med.

[2] Lasala C, Schteingart HF, Arouche N, Bedecarrás P, Grinspon RP, Picard J-Y (2011). SOX9 and SF1 are involved in cyclic AMP-mediated upregulation of anti-Müllerian gene expression in the testicular prepubertal Sertoli cell line SMAT1. Am J Physiol-Endocrinol Metab.

[3] Rey R, Lukas-Croisier C, Lasala C, Bedecarrás P (2003). AMH/MIS: what we know already about the gene, the protein and its regulation. Mol Cell Endocrinol.

[4] Josso N, Picard JY, Rey R, Di Clemente N (2006). Testicular anti-Mullerian hormone: history, genetics, regulation and clinical applications. Pediatr Endocrinol Rev PER.

[5] Ryan BC, Vandenbergh JG (2002). Intrauterine position effects. Neurosci Biobehav Rev.

[6] vom Saal FS (1981). Variation in phenotype due to random intrauterine positioning of male and female fetuses in rodents. J. Reprod. Fertil.

[7] Hacklaender K, Arnold W (2012). Litter sex ratio affects lifetime reproductive success of free-living female Alpine marmots *Marmota marmota*†. Mammal Rev.

[8] Korsoff P, Bogl LH, Korhonen P, Kangas AJ, Soininen P, Ala-Korpela M (2014). A comparison of anthropometric, metabolic, and reproductive characteristics of young adult women from opposite-sex and same-sex twin pairs. Front Endocrinol.

[9] Monclús R, Blumstein DT (2012). Litter sex composition affects life-history traits in yellow-bellied marmots. J Anim Ecol.

[10] Lillie FR (1917). Sex-determination and sex-differentiation in mammals. Proc Natl Acad Sci U S A.

[11] Capel B, Coveney D (2004). Frank Lillie’s freemartin: illuminating the pathway to 21st century reproductive endocrinology. J Exp Zool Comp Exp Biol.

[12] Mishina Y, Behringer RR (1996). The in vivo function of Müllerian-inhibinting substance during mammalian sexual development. Adv Dev Biol.

[13] Padula AM (2005). The freemartin syndrome: an update. Anim Reprod Sci.

[14] Harris RA, Tardif SD, Vinar T, Wildman DE, Rutherford JN, Rogers J (2014). Evolutionary genetics and implications of small size and twinning in callitrichine primates. Proc Natl Acad Sci.

[15] Ross CN, Fite JE, Jensen H, French JA (2007). Demographic review of a captive colony of callitrichids (*Callithrix kuhlii*). Am J Primatol.

[16] Smucny DA, Abbott DH, Mansfield KG, Schultz-Darken NJ, Yamamoto ME, Alencar AI (2004). Reproductive output, maternal age, and survivorship in captive common marmoset females (*Callithrix jacchus*). Am J Primatol.

[17] Chambers PL, Hearn JP (1985). Embryonic, foetal and placental development in the common marmoset monkey (*Callithrix jacchus*). J Zool.

[18] Phillips IR (1975). The embryology of the common marmoset (*Callithrix jacchus*). Adv Anat Embryol Cell Biol.

[19] Rutherford JN, Tardif SD (2008). Placental efficiency and intrauterine resource allocation strategies in the common marmoset pregnancy. Am J Phys Anthr.

[20] Wislocki GB (1939). Observations on twinning in marmosets. Am J Anat.

[21] Benirschke K, Anderson JM, Brownhill LE (1962). Marrow chimerism in marmosets. Science.

[22] Gengozian N, Batson JS, Eide P (1964). Hematologic and cytogenetic evidence for hematopoietic chimerism in the marmoset, *Tamarinus nigricollis*. Cytogenet Genome Res.

[23] Ross CN, French JA, Orti G (2007). Germ-line chimerism and paternal care in marmosets (*Callithrix kuhlii*). Proc Natl Acad Sci U S A.

[24] Goldschmidt B, Moraes IA, Souza LM, Paulino FS, Pissinatti A, Marsico FF (2005). Occurrence of virilixation signals in a female marmoset *Leontopithecus chrysomelas* (Callitrichidae; Primates) with 46, XX/46, XY chimerism. Isr J Vet Med.

[25] Sanchez-Morgado JM, Haworth R, Morris TH (2003). XY female marmoset (*Callithrix jacchus*). Comp Med.

[26] Smith AS, Birnie AK, French JA. Prenatal androgens affect development and behavior in primates. In Building Babies: Primate Development in Proximate and Ultimate Perspective. Edited by Clancy KBH, Hinde, K, Rutherford, JN. New York: Springer; 2013, 103–132.

[27] Sweeney CG, Curran E, Westmoreland SV, Mansfield KG, Vallender EJ (2012). Quantitative molecular assessment of chimerism across tissues in marmosets and tamarins. BMC Genomics.

[28] Ardito G, Lamberti L, Bigatti P, Crovella S, Oberto G (1995). No correlation between chimerism and fertility in Callithrix jacchus (Callithricidae, Primates). Int J Anthropol.

[29] Rutherford JN, Colon DGL, Ross CN, Tardif SD (2014). Developmental origins of pregnancy loss in the adult female common marmoset monkey (*Callithrix jacchus*). PLoS One.

[30] Ahmed SF, Hughes IA (2002). The genetics of male undermasculinization. Clin Endocrinol (Oxf).

[31] Warne GL, Kanumakala S (2002). Molecular endocrinology of sex differentiation. Semin Reprod Med.

[32] Jaquish CE, Tardif SD, Toal RL, Carson RL (1996). Patterns of prenatal survival in the common marmoset (*Callithrix jacchus*). J Med Primatol.

[33] Tardif S, Ross C, Smucny D. Building marmoset babies: trade-offs and cutting bait. In Building Babies: Primate Development in Proximate and Ultimate Perspective. Edited by Clancy KBH, Hinde, K, Rutherford, JN. New York: Springer; 2013, 169–186.

[34] Mustoe AC, Jensen HA, French JA (2012). Describing ovarian cycles, pregnancy characteristics, and the use of contraception in female white-faced marmosets, *Callithrix geoffroyi*. Am J Primatol.

[35] Hiort O (2013). Clinical and molecular aspects of androgen insensitivity. Endocr Dev.

[36] Zhang J (2000). Rates of conservative and radical nonsynonymous nucleotide substitutions in mammalian nuclear genes. J Mol Evol.

[37] Choi Y, Chan AP (2015). PROVEAN web server: a tool to predict the functional effect of amino acid substitutions and indels. Bioinformatics..

[38] Adzhubei I, Jordan DM, Sunyaev SR. Predicting functional effect of human missense mutations using PolyPhen-2. Curr Protoc Hum Genet. 2013;7–20. 10.1002/0471142905.hg0720s76 PMC4480630 23315928

[39] Sim N-L, Kumar P, Hu J, Henikoff S, Schneider G, Ng PC (2012). SIFT web server: predicting effects of amino acid substitutions on proteins. Nucleic Acids Res.

[40] Zielinski WJ, Vandenbergh JG (1991). Effect of intrauterine position and social density on age of first reproduction in wild-type female house mice (*Mus musculus*). J Comp Psychol.

[41] Zielinski WJ, vom Saal FS, Vandenbergh JG (1992). The effect of intrauterine position on the survival, reproduction and home range size of female house mice (*Mus musculus*). Behav Ecol Sociobiol.

[42] Drickamer LC, Arthur RD, Rosenthal TL (1997). Conception failure in swine: importance of the sex ratio of a female’s birth litter and tests of other factors. J Anim Sci.

[43] Hammond GL (2011). Diverse roles for sex hormone-binding globulin in reproduction. Biol Reprod.

[44] Wu T-S, Hammond GL (2014). Naturally occurring mutants inform SHBG structure and function. Mol Endocrinol.

[45] Hammond GL, Miguel-Queralt S, Yalcinkaya TM, Underhill C, Place NJ, Glickman SE (2012). Phylogenetic comparisons implicate sex hormone-binding globulin in “masculinization” of the female spotted hyena (*Crocuta crocuta*). Endocrinology.

[46] Ma CX, Adjei AA, Salavaggione OE, Coronel J, Pelleymounter L, Wang L (2005). Human aromatase: gene resequencing and functional genomics. Cancer Res.

[47] Rochira V, Carani C (2009). Aromatase deficiency in men: a clinical perspective. Nat Rev Endocrinol.

[48] Stratakis CA (2013). An aroma of complexity: how the unique genetics of aromatase (CYP19A1) explain diverse phenotypes from hens and hyenas to human gynecomastia, and testicular and other tumors. J Clin Endocrinol Metab.

[49] Berra M, Williams EL, Muroni B, Creighton SM, Honour JW, Rumsby G (2011). Recognition of 5α-reductase-2 deficiency in an adult female 46XY DSD clinic. Eur J Endocrinol.

[50] Di Marco C, Bulotta AL, Varetti C, Dosa L, Michelucci A, Baldinotti F (2013). Ambiguous external genitalia due to defect of 5-α-reductase in seven Iraqi patients: prevalence of a novel mutation. Gene.

[51] Tsai M-C, Chou Y-Y, Lin S-J, Tsai L-P (2012). A novel SRD5A2 mutation in a Taiwanese newborn with ambiguous genitalia. Kaohsiung J Med Sci.

[52] Makridakis NM, di Salle E, Reichardt JK (2000). Biochemical and pharmacogenetic dissection of human steroid 5α-reductase type II. Pharmacogenet Genomics.

[53] Silver RI, Russell DW (1999). 5 α-Reductase type 2 mutations are present in some boys with isolated hypospadias. J Urol.

[54] Lagarde WH, Blackwelder AJ, Minges JT, Hnat AT, French FS, Wilson EM (2012). Androgen receptor exon 1 mutation causes androgen insensitivity by creating phosphorylation site and inhibiting melanoma antigen-A11 activation of NH2-and carboxyl-terminal interaction-dependent transactivation. J Biol Chem.

[55] Matias PM, Donner P, Coelho R, Thomaz M, Peixoto C, Macedo S (2000). Structural evidence for ligand specificity in the binding domain of the human androgen receptor Implications for pathogenic gene mutations. J Biol Chem.

[56] Belville C, Van Vlijmen H, Ehrenfels C, Pepinsky B, Rezaie AR, Picard J-Y (2004). Mutations of the anti-Mullerian hormone gene in patients with persistent Mullerian duct syndrome: biosynthesis, secretion, and processing of the abnormal proteins and analysis using a three-dimensional model. Mol Endocrinol.

[57] Josso N, Belville C, di Clemente N, Picard J-Y (2005). AMH and AMH receptor defects in persistent Müllerian duct syndrome. Hum Reprod Update.

[58] Nishi MY, Domenice S, Maciel-Guerra AT, Zaba Neto A, da Silva MACP, Costa EMF (2012). Analysis of anti-Müllerian hormone (AMH) and its receptor (AMHR2) genes in patients with persistent Müllerian duct syndrome. Arq Bras Endocrinol Metabol.

[59] Abduljabbar M, Taheini K, Picard J-Y, Cate RL, Josso N (2012). Mutations of the AMH type II receptor in two extended families with persistent Müllerian duct syndrome: lack of phenotype/genotype correlation. Horm Res Paediatr.

[60] Wongprasert H, Somanunt S, De Filippo R, Picard JY, Pitukcheewanont P (2013). A novel mutation of anti-Mullerian hormone gene in persistent Mullerian duct syndrome presented with bilateral cryptorchidism: a case report. J Pediatr Urol.

[61] Isachenko EF, Nayudu PL, Isachenko VV, Nawroth F, Michelmann HW (2002). Congenitally caused fused labia in the common marmoset (*Callithrix jacchus*). J Med Primatol.

[62] Wedi E, Nayudu PL, Michelmann HW (2011). A case report of spontaneous opening of congenitally fused labia in a female common marmoset (*Callithrix jacchus*) followed by pregnancy and birth of twins. J Med Primatol.

[63] Niimi K, Oguchi A, Nishio K, Okano Y, Takahashi E (2015). Congenital malformation of the vaginal orifice, imperforate vagina, in the common marmoset (*Callithrix jacchus*). J Vet Med Sci.

[64] Missler M, Wolff JR, Rothe H, Heger W, Merker HJ, Treiber A (1992). Developmental biology of the common marmoset: proposal for a“postnatal staging”. J Med Primatol.

[65] Abbott DH (1984). Differentiation of sexual behaviour in female marmoset monkeys: effects of neonatal testosterone or a male co-twin. Prog Brain Res.

[66] Abbott DH, Hearn JP. The effects of neonatal exposure to testosterone on the development of behaviour in female marmoset monkeys. Sex, hormones, and behaviour. 1979;62:299–313. 10.1002/9780470720448.ch14 111908

[67] Abbott DH, Hearn JP (1978). Physical, hormonal and behavioural aspects of sexual development in the marmoset monkey, *Callithrix jacchus*. J Reprod Fertil.

[68] Birnie AK, Smith AS, Nali C, French JA (2011). Social and developmental influences on urinary androgen levels in young male white-faced marmosets (*Callithrix geoffroyi*). Am J Primatol.

[69] Dixson AF (1986). Plasma testosterone concentrations during postnatal development in the male common marmoset. Folia Primatol Basel.

[70] Abbott DH, Hearn JP (1979). The effects of neonatal exposure to testosterone on the development of behaviour in female marmoset monkeys. Sex Horm Behav.

[71] Dixson AF (1993). Sexual and aggressive behaviour of adult male marmosets (*Callithrix jacchus*) castrated neonatally, prepubertally, or in adulthood. Physiol Behav.

[72] Dixson AF (1993). Effects of testosterone propionate upon the sexual and aggressive behavior of adult male marmosets (*Callithrix jacchus*) castrated as neonates. Horm Behav.

[73] Epple G, Alveario MC, Belcher AM (1990). Copulatory behavior of adult tamarins (*Saguinus fuscicollis*) castrated as neonates or juveniles: effect of testosterone treatment. Horm Behav.

[74] Perelman P, Johnson WE, Roos C, Seuánez HN, Horvath JE, Moreira MA (2011). A molecular phylogeny of living primates. PLoS Genet.

[75] Wildman DE, Jameson NM, Opazo JC, Soojin VY (2009). A fully resolved genus level phylogeny of neotropical primates (Platyrrhini). Mol Phylogenet Evol.

