# Supplementary material for: Gene changes may minimize masculinizing and defeminizing influences of exposure to male cotwins in female callitrichine primates
Source: Biol Sex Differ. 2016 Jun 2;7:28. doi: 10.1186/s13293-016-0081-y (PMC4890500; doi:10.1186/s13293-016-0081-y)
Supplement: Additional file 1: — Regions of candidate genes sequenced de novo for this study and PCR primers used to amplify selected regions. (DOCX 19 kb) [file 13293_2016_81_MOESM1_ESM.docx]

**Additional file 1. PCR primers used to amplify genomic coding regions of target genes (Underlined primer are the nested primers).**

| Gene | Position/exon | Forward primer (5'-3') | Reverse primer (5'-3') | Amplicon size (bp) |
| --- | --- | --- | --- | --- |
| *SHBG* | 261; exon 5 | SHBG-7F: 5’ AGATTGATGGGGACTCTGTGC 3’  SHBG-8F: 5’ GACTCTGTGCTGCTGAGGGTG 3’ | SHBG-7R: 5’ CTCGGAGATTGAATTCTGCAT 3’ | 536bp, 525bp |
|  | 332; exon 6 | SHBG-9F: 5’ CTGGACAAACAGGCCGAGAT 3’  SHBG-10F: 5’ AGCATCTGCCCCGACTAACC 3’ | SHBG-9R: 5’ CTTGGAGGTAGAGACTGAGCC 3’ | 380bp, 357bp |
|  | 395,398; exon 7 | SHBG-13F: 5’ CTGCAGAAGGTGGTGCTGTCT 3’ | SHBG-13R: 5’ AGTGCCATTGCCTGGGTTCT 3’  SHBG-14R: 5’ ATGGCTTCTGTTCAGGGCTCG 3’ | 596bp, 591bp |
| *CYP19A1* | 185; exon 4 | CYP19A1-185-1F: 5’ TGTGCCCTGGAATTCATGATG 3’  CYP19A1-185-2F: 5’ TGATTCTCTGATGTGCACCCT 3’ | CYP19A1-185-1R: 5’ TCCTGGCCCCTTGTTCAATAA 3’  CYP19A1-185-2R: 5’ GTCCAGAGGCAAGAGTGAAAA 3’ | 286bp, 232bp |
|  | 211; exon 5 | CYP19A1-211-1F: 5’ CAACAAGGATGGCAAGGAGAA 3’  CYP19A1-211-2F: 5’ CACATGGCTAAAACCCCTACTG 3’ | CYP19A1-211-1R: 5’ ACAGCAAAGACACAAGGGTAAA 3’  CYP19A1-211-2R: 5’ AAACAGCAAAGACACAAGGGT 3’ | 399bp, 348bp |
|  | 268; exon 6 | CYP19A1-268-1F: 5’ TCCTAACCAGAACTCTTGGCT 3’  CYP19A1-268-2F: 5’ TGGCAAATAAATCTGTTTCGCT 3’ | CYP19A1-268-1R: 5’ AACATACGTGGCATGGGAATT 3’  CYP19A1-268-2R: 5’ CGTGGCATGGGAATTATGGTT 3 ‘ | 378bp, 222bp |
|  | 436, 466, 499; exon 9 | CYP19A1-436-F1: 5’ ATGAGGGGTGAATCAAACAGG 3’  CYP19A1-436-F2: 5’ CAAAACAGAGGGTTCTGACTG 3’ | CYP19A1-436-R1: 5’ CACTATTGGCAAGGATGGATG 3’  CYP19A1-436-R2: 5’ 'CCAGCCTTCTTTAGTGTTCCA 3’ | 522bp,363bp |
| *SRD5A2* | 2, 28, 41, 49, 64; exon 1 | SRD5A2-1F: 5’ ACATAGGGTGGTGTCTCGCG 3’  SRD5A2-2F: 5’ GTGTCTCGCGCTCCATAAAG 3’ | SRD5A2-1R: 5’ GTTCCTAGGTGCGCGCTTAG 3’  SRD5A2-2R: 5’ TGTAAGCAGAAGAAGCCCAGA 3’ | 461bp, 351bp |
|  | 171, 179; exon 3 | SRD5A2-171-F1: 5’ CCCCTCTTTCATTTTAGCTTA 3’  SRD5A2-171-F2: 5’ AAGCACCAAAAATCTGCACAC 3’ | SRD5A2-171-R1: 5’ GTTGTGACGGGACTGGGTATG 3’  SRD5A2-171-R2: 5’ AAGAGCAAGCTGAGAGCCATT 3’ | 352bp, 288bp |
|  | 214; exon 4 | SRD5A2-214-F1: 5’ GCAGGTGGCATGTTTACGTAT 3’  SRD5A2-214-F2: 5’ TCTGGAGCCAACTTCTTCGGT 3’ | SRD5A2-214-R1: 5’ TGCTCTGCAGGTTAAAAGCCT 3'  SRD5A2-214-R2: 5’ AAAAAGCTACGTGAATGCAAC 3 | 231bp, 173bp |
| *AR* | 58-74, 81, 99, 139, 219; exon 1 | AR-QQ-1F: 5’ CTTTTGCGTGGTTGCTCCTG 3’  AR-QQ-2F: 5’ GTAGATTCAGCCAAGCTCAAGG 3’ | AR-QQ-2R: 5’ TGCCCCCTAAGTAACTGTCCT 3’ | 773bp, 635bp |
|  | 335, 351, 471, 477, 499, 504; exon 1 | AR-AAA-1F: 5’ TTGTGTAAGGCAGTGTCGGTG 3’  AR-AAA-2F: 5’ GCCGAATGCAAAGGTTCTCT 3’ | AR-AAA-1R: 5’ CTGGAAGGAGAAACTTACCGC 3’  AR-AAA-2R: 5’ CCCATCTCGCTTTTGACACA 3’ | 893bp, 686bp |
|  | 685, exon 4 | AR-685-1F: 5’ GGTTTAGCAGGTATTTGGGATG 3’  AR-685-2F: 5’ GTGTTGAATGAGCACTTGTCC 3’ | AR-685-1R: 5’ GCCACATAAGACACCCGATAA 3’  AR-685-2R: 5’ TCCCACAGGGTTATGATGAAG 3’ | 704bp, 571bp |
| *AMH* | 3, 5; exon 1 | AMH-3-5-F: 5’ ‘ TCCGCTTGGCCCACTTAA 3’ | AMH-3-5-R1: 5’ GGAAGGCCTGCTCATAGCTTC 3’  AMH-3-5-R2: 5’ CCGCTGCCATTGCTGTCC 3 | 297bp, 247bp |
|  | 50, 52, 57 in exon 1 | AMH-F1: 5’ CAAACACCCACCTTCCGCTT 3’  AMH-F2: 5’ CAGCCCCTCACATCACCCA 3’ | AMH-R1: 5’ CCCTCATCACAGTGACCTCGG 3’  AMH-R2: 5’ CCAGGGTACAGCACCAGCAG 3’ | 1179bp, 1108bp |
|  | 195; exon 2 | AMH-E2-3-F1: 5’ TTCAATGGCTCAGGTGTTCCC 3’ | AMH-E2-3-R1: 5’ CCGAATAGCAGCGCCTTCAG 3’ | 569bp |
|  | 292, 295; exon 4 | AMH-E4-F1-1: 5’ TCCCGAGACACCCACTACC 3’  AMH-E4-F1-2: 5’ TTGACCCTGCAGCCTCGAG 3’ | AMH-E4-R1: 5’ CGTGAGCGTCTCCAGGAAG 3’ | 494bp, 440bp |
|  | 331, 342, 356, 379, 387, 389, 402, 440, 497; exon 4 | AMH-E5-QF1: 5' TTCCTGGAGACGCTCACGC 3' | AMH-E5-QR1: 5' TTGTTGGCCTGGTAGGTCTCG 3'  AMH-E5-QR2: 5' TCTCGGGGATGAGGACGGA 3' | 587bp, 571bp |
|  | 543; exon 4 | AMH-543-F1: 5’ ‘CGAGCCTTCCGTCCTCATCC 3’  AMH-543-F2: 5’ ‘CGAGACCTACCAGGCCAACAAC 3’ | AMH-543-R1: 5’ CCGGAACATGCAAGGACACC 3’  AMH-543-R2: 5’ GTCACCGGCAGCCACACTC 3’ | 490bp, 245bp |
| *AMHR2* | 13 in exon 1; 57 in exon 2 | AMHR2-F1: 5’ GAAGTCCCACGATGCCCTGTA 3’  AMHR2-F2: 5’ TGCCCTGTATCTGAAGAAAGC 3’ | AMHR2-R1: 5’ AGGCAGATGACTGTAATTGGCA 3’  AMHR2-R2: 5’ GCAGAAGTCAGTGCCACAGGA 3’ | 1015bp, 979bp |
|  | 164 in exon 4; 176, 190, 195 in exon 5; 257, 268 in exon 6 | AMHR2-F3: 5’ GATGCCGAGACAGTGATGAGC 3’  AMHR2-F4: 5’ GTCCCTCCACTGTGACCCAAG 3’ | AMHR2-R3: 5’ TGATGCTCTCACCTTGGGATG 3’  AMHR2-R4: 5’ GGGATGCAGTTCCAGTATTAGC 3’ | 1204bp, 1158bp |
|  | 289, 293; exon 7 | AMHR2-F9-1: 5’ GGGAGATGCAGGGAGAAGACT 3’  AMHR2-F9-2: 5’ CTTGCTCCCTGAAATGGATCA 3’ | AMHR2-R9: 5’ CTCAGATCTCGGTGGGCAAT 3’ | 389bp, 359bp |
|  | 571; exon 11 | AMHR2-571-F1: 5’ GAGCCATCCCTTCCCAGAG 3’  AMHR2-571-F2: 5’ TTCCCAGAGGGCTGTCCAC 3’ | AMHR2-571-R1: 5’ GGCAGTGGTGAGACAGACAAG 3’  AMHR2-571-R2: 5’ CAAGACAGCTATACAATTGCCA 3’ | 267bp, 245bp |
