# Supplementary material for: Gene changes may minimize masculinizing and defeminizing influences of exposure to male cotwins in female callitrichine primates
Source: Biol Sex Differ. 2016 Jun 2;7:28. doi: 10.1186/s13293-016-0081-y (PMC4890500; doi:10.1186/s13293-016-0081-y)
Supplement: Additional file 2: — Tissue or DNA source for gene sequencing. (DOCX 67 kb) [file 13293_2016_81_MOESM2_ESM.docx]

**Additional file 2.** **Animal information for genetic analyses**

| Scientific name | Common name | Sex | DNA source | Institution | Provider | IACUC^1^ |
| --- | --- | --- | --- | --- | --- | --- |
| *Callithrix kuhlii* | Wied's marmoset | 1M, 1F | Tail | University of Nebraska at Omaha | Jeffrey French, Heather A. Jensen | 12-099-12 |
| *Callithrix penicillata* | Black tufted-ear marmoset | 1M, 1F | Tail | University of Nebraska at Omaha | Jeffrey French, Heather A. Jensen | 12-099-12 |
| *Leontopithecus rosalia* | Golden lion tamarin | 2M, 2F | Tail | University of Nebraska at Omaha | Jeffrey French, Heather A. Jensen | Tissue bank |
| *Saguinus midas* | Red-handed tamarin | 1M, 1F | Whole  blood | Southwest National Primate Research Center | Jerilyn Pecotte | 1243SM |
| *Callimico goeldii* | Goeldi’s marmoset | 2M, 2F | Whole blood | Chicago Brookfield Zoo | Mark Warneke, Jay Petersen | Tissue bank |

^1^IACUC, Institutional Animal Care and Use Committee.
